# Progressive Epidural Hematoma in Patients with Head Trauma: Incidence, Outcome, and Risk Factors

**DOI:** 10.1155/2012/134905

**Published:** 2012-12-18

**Authors:** Hao Chen, Yan Guo, Shi-Wen Chen, Gan Wang, He-Li Cao, Jiong Chen, Yi Gu, Heng-Li Tian

**Affiliations:** Department of Neurosurgery, Shanghai sixth People Hospital, Shanghai Jiaotong University, Shanghai 200233, China

## Abstract

Progressive epidural hematoma (PEDH) after head injury is often observed on serial computerized tomography (CT) scans. Recent advances in imaging modalities and treatment might affect its incidence and outcome. In this study, PEDH was observed in 9.2% of 412 head trauma patients in whom two CT scans were obtained within 24 hours of injury, and in a majority of cases, it developed within 3 days after injury. In multivariate logistic regression, patient gender, age, Glasgow Coma Scale (GCS) score at admission, and skull fracture were not associated with PEDH, whereas hypotension (odds ratio (OR) 0.38, 95% confidence interval (CI) 0.17–0.84), time interval of the first CT scanning (OR 0.42, 95% CI 0.19–0.83), coagulopathy (OR 0.36, 95% CI 0.15–0.85), or decompressive craniectomy (DC) (OR 0.46, 95% CI 0.21–0.97) was independently associated with an increased risk of PEDH. The 3-month postinjury outcome was similar in patients with PEDH and patients without PEDH (*χ* ^2^ = 0.07, *P* = 0.86). In conclusion, epidural hematoma has a greater tendency to progress early after injury, often in dramatic and rapid fashion. Recognition of this important treatable cause of secondary brain injury and the associated risk factors may help identify the group at risk and tailor management of patients with TBI.

## 1. Introduction

 Traumatic brain injury (TBI) is a common cause of death and disability, as well as one of the most important health and social problems in the majority of countries including China. A well recognized secondary insult of TBI is epidural hematoma (EDH), which is classically considered to be an acute complication of TBI whose maximum development takes place immediately following trauma [1]. However, there are also many reported cases of delayed/progressive epidural hematoma or very rare chronic epidural hematoma [2–4].

As a special pathological dimension, progressive epidural hematoma (PEDH) is considered in the patient whose repeat computerized tomography (CT) scans revealed new epidural hematoma or an increase in size compared with initial CT finding. Actually, with the development of neuroimaging technology and accessibility, a policy requiring repeat CT scanning was adopted in patients with head injury more than before, and thus it has become apparent that progressive epidural hematoma is indeed common. Although the clinical presentations and radiological findings of PEDH have been known, its significance is uncertain, and the effects of clinical variables on development of PEDH have not been previously described in detail. The aim of this study was to determine the incidence and the timing of PEDH in patients with head trauma, and also to identify the risk factors of PEDH and its impact on outcome.

## 2. Clinical Materials and Methods

### 2.1. Patient Population

A retrospective review of 667 TBI patients admitted to our institution between 2007 and 2009 was performed. Patients delivered within 4 h whose highest abbreviated injury score (AIS) was 3 or less (other than head injury) were considered to be isolated TBI cases and were included. Patients who were considered open or penetrating injury were excluded from the study. For prevention of visitations from interfering factors, patients who had a history of coagulopathy, such as vessel thrombosis or thrombocytopenia, and those on a long-term anticoagulant therapy were also excluded. Those who died in the emergency department before a CT scan was performed, those who deteriorated and died before a second CT scan was performed, and those with incurable conditions were also excluded as having incomplete clinical data. Accordingly, 412 patients were analyzed in this study. Demographic data (gender and age), the time and mechanism of trauma, Glasgow Coma Score (GCS) at admission, and the time stamped on the first CT scan films were documented when the patients arrived at the emergency room. 

### 2.2. Patient Management

 All patients were evaluated with an initial CT scan and were followed with serial neurological examinations. Each patient had obtained CT scans at least 2 times within the first 24 hours postinjury. For some patients with diffuse brain injury, follow-up CT scans were scheduled on the 3rd day, 5th day, and 7th day after trauma. All the imaging studies were technically adequate and reviewed by the staff of the radiology department. The volume of the hematoma was calculated following the formula *A* × *B* × *C* × 0.5, where *A* and *B* represent the largest perpendicular diameters through the hyperdense area on CT scan, and *C* represents the thickness of the lesion [5]. Indications for surgery were a midline shift greater than 5 mm, a large volume of hematoma greater than or equal to 30/10 mL (supratentorial/infratentorial), or neurological deterioration. Decompressive craniectomy (DC) was performed with a large bone flap (diameter > 10 cm) and durotomy centered on the flap, and the enlarged duraplasty with autofascia or artificial materials was performed. Brain tissues were not routinely removed except for cases with severe contusion. Some patients with a GCS of 8 or below had intracranial pressure (ICP) monitoring via fiberoptic intraparenchymal catheters or ventriculostomy. The average daily hours during which ICP readings were higher than 20 mm Hg were calculated. Any clinical deterioration or increase in ICP indicated that another CT scan should be obtained. Patients were evaluated and treated according to the currently accepted international guidelines [6]. 

Blood samples routinely obtained in the emergency room were analyzed for coagulation parameters, including platelet counts (PLT), prothrombin time (PT), activated partial thromboplastin time (APTT), fibrinogen level (Fbg), and the concentration of D-dimer. The International Normalized Ratio (INR) was used as a surrogate for PT to account for institutional variability in the measurement of this marker. Outcome was evaluated using the Glasgow Outcome Scale (GOS) 3 months after the trauma [7]. An unfavorable neurological outcome was defined as a death, vegetative state, or severe disability (GOS = 1, 2, 3).

### 2.3. Defining PEDH and Predictors

We defined PEDH as an appearance of new hematoma(s) or a conspicuous increase in the size of hematoma(s), that is, to a 25% or more increase in at least one dimension of one or more lesions seen on the first postinjury CT scan. In ambiguous cases, the report dictated by the neuroradiologist was used to determine the presence or absence of hemorrhagic progression. Patients in whom the dictated report noted no progression were classified as having a nonprogressive hemorrhage [8]. A diagnosis of PEDH was revised if follow-up studies indicated the findings were artifact or inconsistently visualized. 

Several clinical variables were chosen that might potentially contribute to PEDH (Table 2). Patient age and sex, GCS scores at admission, and the time interval from injury to first CT scan were assessed. Skull fracture, as evidenced by radiologic and neurologic symptoms or signs, was also analyzed. Criteria for TBI-associated early coagulopathy included the presence of thrombocytopenia (PLT < 100,000/mL) and/or elevated INR > 1.2 and/or prolonged APTT > 36 seconds at admission [9, 10]. Early hypotension was defined as a systolic blood pressure (BP) lower than 90 mm Hg and/or diastolic pressure less than 40 mm Hg, documented in the prehospital setting or before the second CT scan was performed. The need for DC was also evaluated as a possible predictor of progressive epidural hemorrhage.

### 2.4. Statistical Analysis

The data were analyzed using Excel (Microsoft Corp., Redmond, WA) and SAS software (Version 8.1, SAS Institute, Cary, NC). Descriptive techniques, such as frequency tables and whisker plots, were used to evaluate the association. Univariate analysis for each variable was assessed using *χ* ^2^ test or, where appropriate, the Fisher's exact test. A multivariate logistic regression analysis was performed to determine the independent predictors of PEDH. Predictors were defined as being significant (*P* < 0.05) after all predictors were added to the model. The outcome variable was dichotomized as PEDH positive or negative.

## 3. Results

 412 patients with head trauma in admission were the focus of this analysis. The patients' age ranged from 12 to 86 years, with mean age of 35.4 years; 251 (61%) patients of the series were male and 161 (39%) were female. The mechanisms of trauma included 273 (67.1%) motor vehicle collisions, 82 (19.9%) falls, 23 (5.6%) heavy strikes (patients who were hit by heavy objects such as bricks, sticks, or falling objects), and 34 (8.3%) cases of assaults. Mean GCS upon admission was 8.2 (range, 3–15). In addition, 412 patients underwent a total of 2765 repeat CT scans. Mean time from injury to first CT scan was 2.7 hours and the time between first and second CT scans was 8.7 hours. 154 patients underwent decompressive craniectomy due to associated intracranial contusions and hematoma and severe cerebral swell. Monitoring of ICP was conducted in 103 patients with a GCS of 8 or below. 

**Figure 1:**
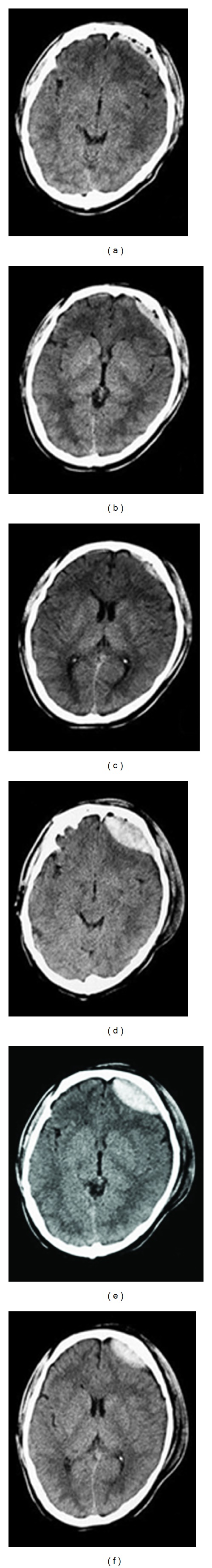
Case 1. Progressive epidural hematoma in a 32-year-old man after motor vehicle collisions. (a), (b), and (c): Initial CT image obtained 2 hours after-injury, demonstrating a small epidural hematoma in the left frontal. (c), (d), and (e): The second CT scan obtained 6 hours after-injury revealed an unambiguous increase in hematoma size.

**Figure 2:**
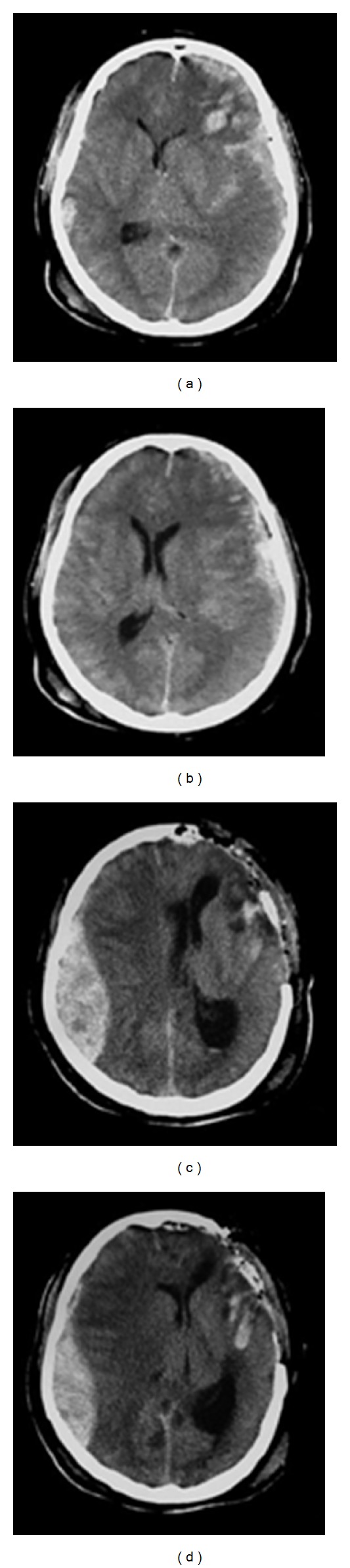
Case 2. Progressive epidural hematoma in a 51-year-old man after motor vehicle collisions. (a) and (b): Initial CT scans showing a left acute subdural hematoma with ventricular compression and midline shift to the right. (c) and (d): Repeat CT scans 16 hours aftertrauma and 13 hours after left frontal-temporal craniectomy; a contralateral epidural hematoma is revealed.

 Among the 412 patients evaluated, thirty eight (9.2%) developed PEDH during their hospitalization. At admission, these patients had no epidural hematoma in 23 cases, small epidural hematoma in 15 cases. Ages of these patients ranged from 23 to 72 years and average 33.5 years. The timing of PEDH development varies from 2 hours to 7 days and average 23 hours. Progressive epidural hematoma appeared after injury within the first 6 hours in 11 (29.0%) cases, 7 hours to 24 hours in 14 (36.8%) cases, one day to 3 days in 10 (26.3%) cases and 3 days to 7 days in 3 (7.9%) cases. On average, patients with PEDH tended to undergo ICP monitoring for a longer time than patients without PEDH (5.0 ± 3.4 days versus 3.6 ± 2.5 days, *P* = 0.03). Additionally, in patients with PEDH there were more hours per day when ICP was higher than 20 mm Hg than in patients without PEDH (3.5 ± 2.0 versus 2.3 ± 1.5, *P* = 0.002). Of the 38 patients with PEDH, surgical evacuation of the progressive hematoma was performed on 30(79%). At 3 months after the injury, an unfavorable neurological outcome was seen in 36.8% of patients with PEDH and in 39% of those without PEDH, respectively (Table 1, *χ* ^2^ = 0.07, *P* = 0.86). Figures 1 and 2 show sequential CT scans obtained in two cases to illustrate the definition of PEDH in this investigation.

**Figure 3:**
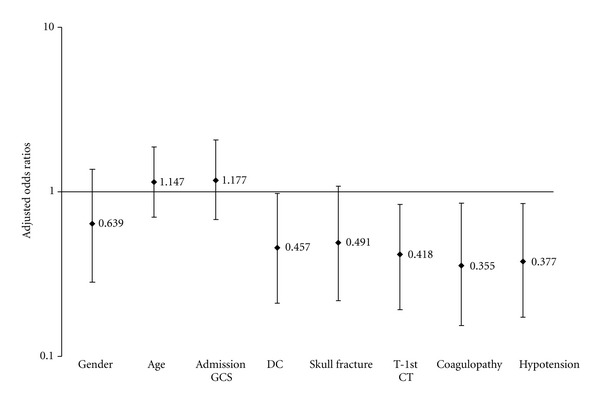
Adjusted odds ratios for PEDH risk factors, where DC is decompressive craniectomy and T-1st CT is time interval from trauma to 1st CT.

 Univariate analysis revealed a significant correlation between occurrence of progressive epidural hematoma and time from injury to 1st CT, low systolic BP, skull fracture, and DC. Gender, age, and admission GCS score did not influence the development of PEDH (Table 2). The results of multivariate logistic regression are summarized in Table 3. Decompressive craniectomy (OR 0.46, 95% CI 0.21–0.97), time from injury to 1st CT (OR 0.42, 95% CI 0.19–0.83), the presence of coagulopathy (OR 0.36, 95% CI 0.15–0.85), and early hypotension (OR 0.38, 95% CI 0.17–0.84) were each independent predictors of PEDH. There was no independent association between PEDH and patient age, gender, GCS score at admission and skull fracture. Odds ratios are adjusted for all of the above variables (Figure 3). 

## 4. Discussion

 A policy of routine follow-up CT imaging for patients with head injury is commonly adopted at many trauma centers. It is well recognized that early repeated CT scanning is potentially crucial for detecting progressive hemorrhage, and triggering timely medical or surgical intervention to avert the developing of serious secondary insults in patients with TBI. Furthermore, CT scanning has revealed that delayed/progressive hematomas after head trauma are more common than had been previously suspected. Several studies have reported that early progressive hemorrhage occurred in approximately 30–42.3% of head injured patients, and it occurs most frequently in intraparenchymal contusion or hematoma [11–13].

Progressive epidural hematoma is also often observed on serial CT scans in head trauma patients. It is currently defined on the basis of a radiologic criterion: epidural hematoma that is not present, or only small size in the first CT scan after trauma, but that appears, or significant enlargement in hematoma size in sequential repeat CT scan during patient evolution. However, the amount of increasing hematoma and the timing of presence of hemorrhagic progression are ambiguous. In our study, PEDH was defined as an appearance of new hematoma, or a 25% or more unequivocal increase in the size of hematoma during hospitalization. The reported incidence of delayed epidural hematoma varies from 5.6% to 13.3% [8, 14, 15]. Similarly, we observed progressive epidural hematoma in 9.22% of head-injured patients.

In the present study, PEDH mainly developed early during the clinical course, and twenty-five patients developed PEDH within the 1 day after injury. Later than 3 days after injury, PEDH was rare. However, in some setting epidural hematoma occasionally develops more slowly. In our study, a 39-year-old male patient had significant increases in the size of the epidural hematoma 7 days after trauma and ultimately required surgery. Borovich et al. have reported a patient with an interval of 11 days between head injury and diagnosis of delayed EDH [16]. 

We found that the average age of the patients with PEDH was younger than the patients without PEDH in this series, although there was no significant association between age differential and PEDH by statistical analysis. It has been hypothesized that dural-based vessels might be more easily torn or avulsed due to deformation of the skull in younger patients, because the dura becomes increasingly adherent to the skull with advanced age, which reduces the risk of epidural hematoma. Although the results showed no relation between the GCS score and PEDH, this scale is useful to evaluate the status of patients with TBI and to improve outcomes. Ono et al. retrospectively analysed 272 patients with severe head injury and suggested the GCS score was the only significant outcome prognostic factor in the EDH group [17]. 

 The timing of the first CT scan is clearly an important factor to predict the occurrence of progressive epidural hematoma. It is not doubt that the sooner after injury the first scan is performed, the greater likelihood of subsequent hemorrhagic progression on later CT scans is. A similar finding was reported by Oertel et al. In their study of 107 patients in whom the initial CT scan was performed within the first 2 hours after-injury, 48.6% had progressive hemorrhagic injury, compared with 22.9% of patients who underwent their injury CT scan within 2–10 hours after-injury [8]. It is possible that head trauma patients undergo their first cranial CT scan, It is possible that head trauma patients undergo their first cranial CT scan, if the time interval from trauma to the initial CT scan is shorter, the epidural hematoma formation does not achieve near-maximum size. During this initial period, the intracranial injury may be rapidly evolving. Additionally, following repeat CT can reveal an increase in hematoma size compared with initial CT finding. 

Another significant finding in the present study was that decompressive craniectomy seemed to be independently predictive of PEDH. Postoperative intracranial hematomas had been observed in previous reports with an incidence ranging from 7.8% to 61% [18]. In the present study, we found that almost 16% of patients who underwent DC showed significant epidural hemorrhage progression. Decompressive craniectomy has been proved to be a useful means to decrease intracranial pressure, and usually performed as a last resort in patients with malignant edema because of intracranial hematomas. Whilst technically straightforward, the procedure is not without significant complications. However, a sudden decrease of cerebral vessels pressure after DC may occasionally cause progressive hemorrhage [19]. Mohindra et al. reported two cases of contralateral extradural haematoma after an earlier DC for acute subdural haematoma [20]. Su et al. also reported 12 traumatic patients with acute subdural hematoma who developed delayed contralateral EDH after hematoma evacuation, and only three patients with less severe head injury had good recovery [21]. Thus, we proposed that routine postoperative CT scan should be performed immediately after cranial surgery for head trauma, particularly in those with a skull fracture contralateral to the original hematoma. This would help in timely detection and treatment of such a complication. 

The fact that skull fracture was not the predictor of PEDH was somewhat surprising. The relationship between skull fracture and an increased incidence of epidural hematoma or delayed epidural hematoma has been established in previous studies [22, 23]. Piepmeier and Wagner have reported that the overall incidence of skull fractures in the patients with intracranial hemorrhages was 48%, while in those with delayed hematomas it was at least 75% [22]. Moreover, some authors believed that presence of a skull fracture has been identified as a common feature of reported cases of delayed epidural hematoma and should be considered a predisposing factor for the development of this complication [21, 24]. However, we found in this series that skull fracture alone was not a predictive factor of PEDH by the multivariate analysis. Considering its retrospective, nonrandomized nature, the results of ours study cannot be completely examined the influence of skull fracture on the PEDH. Further studies are urgently needed.

The three parameters (PLT, PT, and APTT) used to define coagulopathy in this study are available at most of hospitals, yet their reliability in predicting PHI is controversial. Our data suggested that hemocoagulative disorder after injury was an independent risk factor for suffering PEDH. Studies by Stein et al. and Engström et al. also showed that an increase in PT and APTT and a decrease in platelets were predictive for PHI [25, 26]. However, other studies indicate that progressive hemorrhage after head injury is associated with diffuse intravascular coagulation as defined by increased concentration of fibrin degradation products or D-dimer and low fibrinogen concentration [27, 28]. These parameters may serve as an early marker of coagulopathy. Our previous study indicated that a D-dimer level of 5.00 mg/L was considered the cutoff point to prognose the possibility of PHI, with a sensitivity of 72.8% and a specificity of 78.8% [28]. 

Concurrent systemic traumatic lesions leading to a hypotensive state have been classically identified as a mechanism responsible for the delayed appearance of the epidural hematoma, a “tamponade” effect [2]. Nevertheless, our analysis showed such a direct correlation of PEDH with hypotension. We speculated that resuscitation efforts and catecholamine administration in the emergency room could lead to significant variations of BP after initial hypotension, which might increase the risk of rebleeding. Similarly, Borovich et al. reported a case who was hypotensive at the time of emergency evacuation of an epidural hematoma. Blood pressure recovered soon after, and a contralateral delayed traumatic epidural hematoma was demonstrated within 24 hours [16]. A patient reported by Cervantes was initially hypotensive after trauma and PEDH was diagnosed after the patient had undergone an exploratory laparotomy because of a hemoperitoneum [29]. Hence, the effects of hypotension on the development of PHI remain questionable and should be clearly defined further.

In addition, patients with PEDH on average had more hours of elevated ICP during the acute injury period than those without PEDH, in spite of intensive treatment for ICP. By contrast, the outcome followup at 3 months afterinjury did not appear to be affected by the presence of PEDH. Nonetheless, we still strongly recommend that early follow-up CT scans and intensive monitoring are warranted in moderately and severely head injured patients whose initial CT scan demonstrates evidence of an intracranial hemorrhagic injury.

Our study has several limitations. Foremost is the patient-selection bias intrinsic to all retrospective studies. Specifically, the decisions to obtain repeated CT scans in this study were made by the neurosurgeons involved in the care of the individual patients. Almost all such scans were obtained, not through a prospective plan to routinely perform follow-up scans. PEDH was diagnosed according to the repeated CT scan comparing with the first one. Many mildly injured patients had been excluded for the lack of further clinical data including repeated CT scan, so we cannot be sure of its true incidence. Furthermore, more detailed outcome measures could not be effectively collected in our study because of the retrospective design and difficulties with followup in our patient population. Finally, not all the patients received ICP monitoring; therefore data on ICP was incomplete and excluded from the multivariate model. 

## 5. Conclusions

 We performed serial CT scans on 412 in hospital patients with head trauma. Progressive epidural hematoma was encountered in 38 (9.2%), which can be developed within the first three days after head trauma. Early identification and successful management of this complication require a high index of clinical suspicion. The present study demonstrated that low systolic BP, the presence of coagulopathy, decompressive craniectomy, and a shorter time interval between injury and the first CT scan were the risk factors for the development of PEDH. Patients with PEDH have a greater degree of ICP elevations, and almost 80% of those patients require craniectomy for hematoma removal. Based on these findings, we recommend routine follow-up CT scans be done immediately for all patients who deteriorate, or at 12 to 24 hours after admission if there is hypotension or coagulopathy. 

## Figures and Tables

**Table 1:** Association between PEDH and Glasgow Outcome Scale scores.

GOS score at 3 months	No. of patients	PEDH (%)		*P* value
		Positive, *n* = 38	Negative, *n* = 374	
Unfavorable outcome (GOS = 1, 2, 3)	160	14 (36.8)	146 (39)	0.86
Favorable outcome (GOS = 4, 5)	252	24 (63.2)	228 (61)	

GOS: Glasgow Outcome Score; PEDH: progressive epidural hematoma.

**Table 2:** Clinical variables related to the development of PEDH in the patients with head trauma.

Clinical variables	No. of patients	with PEDH	*P* value
Gender			0.6
male	251	25 (10%)	
female	161	13 (8.1%)	
Age (yrs)			0.25
<20	141	9 (6.4%)	
20–40	176	17 (9.7%)	
>40	95	12 (12.6%)	
Admission GCS scores			0.87
13–15	62	5 (8.1%)	
9–12	215	19 (8.8%)	
3–8	135	14 (10.4%)	
Decompressive craniectomy			0.001^b^
Yes	154	24 (15.6%)	
No	258	14 (5.4%)	
Skull fracture^c^			0.000^b^
Yes	132	23 (17.4%)	
No	280	15 (5.4%)	
Time from injury to 1st CT			0.001^b^
<2 hours	193	28 (14.5%)	
2–6 hours	158	9 (5.7%)	
>6 hours	61	1 (1.6%)	
Coagulopathy^c^			0.004^b^
Yes	67	13 (19.4%)	
No	345	25 (7.3%)	
Hypotension^c^			0.000^b^
Yes	74	16 (21.6%)	
No	338	22 (6.5%)	

PEDH: progressive epidural hematoma; GCS: Glasgow Coma Score; CT: computerized tomography.

^a^ *χ* ^2^ test *P* < 0.05 difference between groups.

^b^ *χ* ^2^ test *P* < 0.01 difference between groups.

^c^According to the definition outlined earlier.

**Table 3:** Multivariate logistic regression analysis of the association between risk factors and PEDH.

Clinical variables	OR value	95% CI	*P *value
Gender	0.64	0.28–1.37	0.26
Age (yrs)	1.15	0.70–1.88	0.58
GCS score at admission	1.18	0.68–2.06	0.56
Decompressive craniectomy	0.46	0.21–0.97	0.04
Skull fracture	0.49	0.22–1.08	0.08
Time from injury to 1st CT	0.42	0.19–0.83	0.02
Coagulopathy	0.36	0.15–0.85	0.02
Hypotension	0.38	0.17–0.84	0.02

OR: odds ratio; CI: confidence interval; GCS: Glasgow Coma Score; CT: computerized tomography.
